# The Effect of Using Technology Supported Material in Teaching English to First-Year Primary School Children: On Their Academic Success During COVID-19

**DOI:** 10.3389/fpsyg.2021.756295

**Published:** 2021-10-15

**Authors:** Fatma Köprülü

**Affiliations:** Department of Educational Administration and Supervision, Near East University, Nicosia, Cyprus

**Keywords:** foreign language teaching, academic success, technology-supported English teaching, COVID-19, primary education

## Introduction

Rapid technological developments have brought about the need for more innovative teaching techniques due to changes in applications and the tools used for this purpose. During the recent COVID-19 pandemic, schools and universities were closed and relationships among individuals were minimized (Gupta and Goplani, 2020). Karalis and Raikou (2020) outline that in this context learners experience negative feelings, stress, and anxiety. Sprang and Silman (2013) have pointed out that learners isolated in quarantine have a higher level of possible stress and adjustment disorder and suffer from worries. Many learners at home have experienced emotional and psychological problems during the COVID-19 closures and have failed to communicate actively or cannot be productive (Petrie, 2020). Online learning programs offer learners great opportunities to learn with more convenience AND ONLINE tutoring is one of the MOST prevalent educational tools used around the world during the pandemic (Kao and Chou, 2018).

During the pandemic, many changes in online learning have been observed. Today, technology in the form of computers, laptops, and electronic boards, etc., have replaced traditional teaching approaches using pens, notebooks, and the blackboard. Sen (2011) emphasized that reaching information has become much easier and technology plays a great role in effective and fast outcomes. Karakaya (2013) pointed to the need for teachers to carry out teaching *via* technology and strict controls, otherwise, the young generation will not benefit from rapid developments in technology.

In their studies on the widespread use of and easy access to technology, Günüç et al. (2013) and Ahmadi and Reza (2018) outline that as learners develop skills in the use of technology, they will have to adapt to new responsibilities and this will significantly affect their behaviors.

One of the most effective ways of using technology productively in teaching is to provide material, particularly technological material, which has a significant place in teaching. Sen and Sentürk (2014) stress that use of technology and computers have become a must without resorting to any traditional methods.

Besides providing controlled learning with clear aims for the learners, technology in education helps teachers overcome possible problems and provide a learning environment with suitable, well-designed, and well-prepared materials (Demirel and Yagci, 2012).

Language is the most effective way of communication in an individual's reflecting feelings, thoughts, and wishes.

Concretizing abstract concepts in language teaching is crucial for learners (Karadayı-Taşkıran et al., 2015), as it means they have understood complex ideas. Therefore, supporting tools are necessary for teaching concepts. Studies by both Sirakaya (2015) and Taşkiran et al. (2015) argue that researched reality helps learners become more active participants in learning compared to ordinary lesson materials.

Technological material helps teachers take over tasks and adds to teaching directly. The material in foreign language teaching does not only support learning but also increases interest and helps permanent learning. Proper use of material lessens problems such as unwillingness to learn and indifference to the subject. The internet functions as a virtual library and hence makes authentic language material available to language learners across the globe (Imtiaz et al., 2021).

Kaya (2006) stresses that the material chosen should respond to learners' needs and that learners should be encouraged to design material. DePew similarly (DePew, 2015) has propounded that learners demonstrate a positive attitude toward accessing online language learning material. Therefore, it can be claimed that technological material motivates learners, keeps them alert in class for longer, facilitates achieving targets specified in teaching programs, and helps to sustain permanent learning (Mert and Sen, 2019).

## Problem Statement

Changes in technology affect education to a great extent. In our world today, English has become an essential language, which can easily be taught through the rapid developments in technology in an enjoyable way. Using suitable material and tools while teaching affects learning. Teachers working with primary first-years should closely follow developments in technology and share material and tools with learners to increase quality. Taking up the idea that the teacher should consider learners' needs, their learning time, and provide a suitable teaching environment, this study examined the effect of using technology and material in teaching English to primary first-year learners.

## Aims of the Study

This study aimed to specify the effect of technology-supported material in teaching English to primary first-year learners and their academic success. In fall 2020–21 attendance was on rotation bases in the TRNC (Turkish Republic of North Cyprus). Students would receive face-to-face lessons on 1 day and online on the other. This study aimed to specify any significant difference between these two approaches as well as any permanent learning.

## Methodology

### Research Method

To get an answer to the subject question, an experimental uncensured pre/post-test, one of the quantitative research methods, was conducted to the test-controlled group. Experimental researchers are composed of studies to determine the effect of differences specified by the researcher on dependent variables (Büyüköztürk et al., 2016).

### Participants and Sampling

The participants were randomly selected primary first-year learners from two primary schools in Lefkoşa in the fall 2020–2021 academic year.

### Data Collection

The primary first-year children were given a pre-test before presenting the topic to elicit information about the “My Family” unit, conducted in 5 h throughout the week for consolidation. While the control group had face-to-face lessons through traditional methods, teaching techniques and the material was used with the experimental group.

A post-test was conducted to determine the effectiveness of the methods. To specify the effect of the methods on permanent learning, a “permanency test” was conducted 6 weeks after the post-test without informing the learners. As an experimental model, control and experimental groups were formed. Their prior knowledge was specified through pre/post-tests. Traditional teaching methods were tried with the control groups. The same process was conducted with experimental groups using technology and material. To determine the effect of the methods on academic success, the learners were given a post-test. After 6 months, without informing the learners, they were given a permanent test to determine permanent knowledge through the applied methods.

### Data Analysis

A dependent sampling *t*-test was given to the primary first-year learners and the control group to determine any significant difference between their pre/post, and permanent scores.

To specify any difference in the progress test scores of both groups, a dependent sampling *t*-test with two choices, and the ANOVA test with more than two choices were conducted.

### Findings

As seen in Table 1, 4 learners (20.0%) in the experimental group had a very low, 8 (40.0%) low, 8 (40.0%) average, 1 (5.0) learner in the control group had very low, 10 (50.0%) low, 7 (35.0%) average, and 2 (10.0%) low scores.The findings from the pre-test of the experimental group in terms of gender, 1 (9.0%) girl had very low, 6 (54.6%) girls had low, and 4 (36.4) female students had average scores. None of the female learners had a very good score. In total, 3 (33.3) male students had very low, 2 (22.3) had low, and 4 (44.4) average scores. The post-test scores by the control group concerning any of the devices do not indicate a different effect. The permanent-test results by the control group concerning owing any of the devices do not indicate a different effect. Overall, 1 (10.0%) of the girls in the pre-test control group had very low, 4 (40.0%) low, and 5 (50.0%) average scores. Then, 6 (60.0) of the boys in the pre-test control group had low, 2 (20.0%) average and 2 (10.0) had good scores. At this point, it is clear that in terms of pre-test success scales, the control group showed a higher performance than the experimental group. In terms of gender, in both groups, girls exhibited a lower performance than boys.

**Table 1:** The pre-test success scales, the post- test success scales and permanent test success scales of primary school 1st grade experimental and control group students.

**Test**	**Groups**	**Scales**	**Total**		**Gender**			
					**Girls**		**Boys**	
			***N***	**%**	***N***	**%**	***N***	**%**
Pre-test	Experimental group	Very Low	4	20.0%	1	9.0%	3	33.3%
		Low	8	40.0%	6	54.6%	2	22.3%
		Average	8	40.0%	4	36.4%	4	44.4%
		Good	0	0.0%	0	0.0%	0	0.0%
		Very Good	0	0.0%	0	0.0%	0	0.0%
	Control group	Very Low	1	5.0%	1	10.0%	0	0.0%
		Low	10	50.0%	4	40.0%	6	60.0%
		Average	7	35.0%	5	50.0%	2	20.0%
		Good	2	10.0%	0	0%	2	20.0%
		Very Good	0	0.0%	0	0.0%	0	0.0%
Post-test	Experimental group	Very Low	0	0.0%	0	0.0%	0	0.0%
		Low	0	0.0%	0	0.0%	0	0.0%
		Average	0	0.0%	0	0.0%	0	0.0%
		Good	6	30.0%	5	45.5%	1	11.1%
		Very Good	14	70.0%	6	54.5%	8	88.9%
	Control group	Very Low	0	0.0%	0	0.0%	0	0.0%
		Low	1	5.0%	1	10.0%	1	0.0%
		Average	7	35.0%	3	30.0%	4	40.0%
		Good	7	35.0%	3	30.0%	4	40.0%
		Very Good	5	25.0%	3	30.0%	2	20.0%
Permanent test	Experimental group	Very Low	0	0.0%	0	0.0%	0	0.0%
		Low	0	0.0%	0	0.0%	0	0.0%
		Average	1	5.0%	1	9.1%	0	0%
		Good	7	35.0%	4	36.4%	3	33.3%
		Very Good	12	60.0%	6	54.5%	6	66.7%
	Control group	Very Low	0	0.0%	0	0.0%	0	0.0%
		Low	4	20.0%	2	20.0%	2	20.0%
		Average	7	35.0%	3	30.0%	4	40.0%
		Good	6	30.0%	4	40.0%	2	20.0%
		Very Good	3	15.0%	1	10.0%	2	20.0%

In total, 6 (30.0%) learners of the experimental group had good, and 14 (70.0%) had the highest scores in the post-test. Moreover, 1 (5.0%) of the control group had low, 7 (35.0%) average, 7 (35.0%) good, and 5 (25.0%) had the highest results. In the same test, 5 (45.5) girls scored good and 6 (54.5%) the highest. One (11.1%) of the boys had good, and 8 (88.9%) had the highest scores. The result of the analysis indicates that 1 (10.0%) girl had low, 3 (30.0%) average, 3 (30.0%) good, and 3 (30.0%) the highest scores. Overall, 4 (40.0%) of the boys had average, 4 (40.0%) good, and 2 (20.0%) had the highest scores. The overviewed of these performance scales indicates that the control group had a lower performance than the experimental group. In terms of gender, it was observed that boys had higher scores compared to girls.

The experimental group permanent-test results, as shown in Table 1, are 1 (5.0%) average, 7 (35.0%), and 12 (60%) highest. As for the control group, 4 (20%) had low, 7 (35%) average, 6 (30.0%) good, and 3 (15.0%) highest scores. The analysis result of the permanent-test reveals that 1 (9.1%) girl participant had average, 4 (36.4%) good, and 6 (54.5%) highest scores. In total, 3 (33.3%) of the boys had good, and 6 (66.7%) the highest scores. The result of the analysis showed that 2 (20.0%) of the girls had low, 3 (30.0%) average, 4 (40.0%) good, and 1 (10%) the highest score. Moreover, 2 (20%) of the boys had low, 4 (40%) average, 2 (20.0%) good, and 2 (20%) the highest scores. These performance scale overviews show that the control group had lower scores compared to the experimental group. In terms of gender, in the experimental group boys had higher, but in the control group girls had higher scores.

As shown in Table 2, there is a significant difference (*p* < 0.01) between the averages of the Pre/Permanent-test scores. Moreover, a significant difference between the post-test scores can be observed in the Table (*p* < 0.01). In the post/Permanent-test results a significant difference was observed (*p* < 0.01) in the Post-Permanent-test results.

**Table 2:** The pre/permanent-test and the post/permanent-test results by both the experimental and control groups.

**Groups**	**Test**	***N***	** $\bar{X}$ **	**Ss**	**sd**	***t***	***p***
Experimental	Pre-test	20	42,500	14,001	19	−13,379	0.000
	Permanent-test	20	84,250	8,626			
Control	Pre-test	20	46,000	14,104	19	−3,264	0.004
	Permanent-test	20	62,500	16,741			
Experimental	Post-test	20	88,000	8,335	19	1,370	0.186
	Permanent-test	20	84,250	8,626			
Control	Post-test	20	70,750	12,383	19	1,849	0.080
	Permanent-test	20	62,500	16,741			

## Data Availability Statement

The original contributions presented in the study are publicly available. This data can be found here: https://osf.io/wk467/.

## Ethics Statement

The studies involving human participants were reviewed and approved by Near East University Ethical Committee. Written informed consent to participate in this study was provided by the participants' legal guardian/next of kin.

## Author Contributions

FK wrote the initial abstract, manuscript, and table drafts.

## Conflict of Interest

The author declares that the research was conducted in the absence of any commercial or financial relationships that could be construed as a potential conflict of interest.

## Publisher's Note

All claims expressed in this article are solely those of the authors and do not necessarily represent those of their affiliated organizations, or those of the publisher, the editors and the reviewers. Any product that may be evaluated in this article, or claim that may be made by its manufacturer, is not guaranteed or endorsed by the publisher.
