# Supplementary material for: The Effect of Using Technology Supported Material in Teaching English to First-Year Primary School Children: On Their Academic Success During COVID-19
Source: Front Psychol. 2021 Oct 15;12:756295. doi: 10.3389/fpsyg.2021.756295 (PMC8554301; doi:10.3389/fpsyg.2021.756295)
Supplement: Supplementary file 1 [file Table_1.DOCX]

**Findings**

**Gender of the participants**

**Table1.** **Gender of the primary first-year learners**

**Classes Experimental Control Total**

**f % f % f %**

1st year girls 11 %55.0 10 %50,0 21 %52,5

1st year boys 9 %45,0 10 %50,0 19 %47,5

As in Table 1, 11 (%55) of the participants out of 20 were girls and %9 (45) were boys. As for the control group, 10 (%50) out of 20 were girls and 10 (%5o) were boys.

**Table 2.** **Academic progress t-test results of the experimental group in terms of gender**

**Test Gender f $\bar{X}$ Ss sd t p**

**Pre-test Boys 9 41,111 16,729 18 -,392 ,700**

**Experiment Girls 11 43,636 12,060**

**Post test Boys 9 87,222 8,700 18 -,369 ,717**

**Experiment Girls 11 88,636 8,393**

**Permanent test Boys 9 80,555 9,166 18 ,083**

**Experiment Girls 11 87,272 7,198 1,838**

As can be observed in Table 2, a significant difference in test results between the two groups was not observed (p<0.05) in terms of gender. As for the post and permanent tests are given to the experimental group, a difference in terms of gender was not observed (p<0.05).

The findings do not indicate any significant difference in the scores in pre/post-test by the experimental group in terms of gender. The results in the permanent test showed no significant difference.

**Table 3. Academic success t-test results by the control group in terms of gender.**

**Test Gender N $\bar{X}$ Ss sd t p**

**Pre-test Boys 10 54,444 14,240 18 2,835 ,011**

**Control Girls 10 39,090 9,954**

**Post test Boys 10 73,888 10,833 18 1,027 ,318**

**Control Girls 10 68,181 13,467**

**Permanent test Boys 10 62,777 16,029 18 ,065 ,949**

**Control Girls 10 62,272 18,078**

As it is revealed in Table 3, a significant difference was not observed between the experimental and control groups in the subject question in terms of gender (p<0.05).

The results obtained from the control group indicate that in terms of prior knowledge, girls are more successful than boys.

The findings also show that gender factor has no effect on the scores of the experimental group in the post-test. The same findings apply to the results scored in the permanent test in terms of gender factor.

**Table 4. The case of owning a laptop, computer, or Ipad**

**Total Group Gender**

**Experimental Control Girls Boys**

**Group Group**

**N % N % N % N % N %**

**Yes 13 % 32,5 6 %30,0 7 %35,0 2 %9,5 11 %57,9**

**No 27 %67,5 14 %70,0 13 %65,0 19 %90,5 8 %42,1**

As Table 4 shows, out of 40 learners, 23 (%32.5) owned a laptop, a computer, or an Ipad, whereas 27 (%67.5) did not.

6 (%30.0) of the experimental group owned one of these electronic devices, but 14 (%70.0) did not. Almost the same case applies to the control group, 7 (%30.0) owning the devices, but 13 (%65.0) without any.

Regarding owning the devices in question, the experimental and control groups do not lack any, but there is a significant difference in the subject matter in terms of gender.

**Table 5. The effect of owning an electronic device on academic success in t-test**

**Test Ownership N $\bar{X}$ Ss sd t p**

**Pre-test Yes 6 40,000 16,124 18 -0,512 0,614**

**Experimental No 14 43,571 13,506**

**Post-test Yes 6 87,500 10,368 18 -0,171 0,866**

**Experimental No 14 88,214 7,747**

**Permanent-test Yes 6 82,500 6,123 18 -0,583 0,566**

**Experimental No 14 85,000 9,607**

The pre/post and permanent-test findings showed no significant difference in terms of owning any of the devices (p<0.05). When the scores in the pre-test are overviewed, lacking any of the equipment did not have a different effect on the experimental group. The same findings apply to the post and permanent-test results by the learners. When the post-test results of the experimental group concerning owing any of the devices, a significant difference is not noted.

The permanent-test results by the experimental group concerning owing any of the devices, do not indicate a different effect on their permanent-test scores.

**Table 6. The effect of owning electronic equpiment on academic success t-test**

**Test Ownership N $\bar{X}$ Ss sd t p**

**Pre-test Yes 6 47,500 6,892 18 0,303 0,764**

**Control No 14 45,357 16,462**

**Post-test Yes 6 70,833 16,557 18 0,019 0,984**

**Control No 14 70,714 1,894**

**Permanent Yes 6 60,833 17,151 18 -0,284 0,779**

**Test Control No 14 63,214 17,165**

According to the findings in Table 6, the analysis of the pre/postand permanent –test results does not indicate to any significant difference in terms of owning any of the equipment (p<0.05).

The post-test results by the control group concerning owning any of the devices, do not indicate to a different effect.

The permenent-test results by the control group concerning owning any of the devices,do not indicate to a different effect.

The same findings apply to control group in their pre/post and permanent-test results in which a significant difference was not observed.

**Table 7. The Pre-test success scale of the experimental and control groups**

**Test Groups Scales Total Gender**

**Girls Boys**

**N % N % N %**

**Pre-test Experimental Very low 4 %20,0 1 %9,0 3 %33.3**

**Group Low 8 %40,0 6 %54,6 2 %22,3**

**Average 8 %40,0 4 %36,4 4 %44,4**

**Good 0 ,0% 0 ,0% 0 ,0%**

**Very good 0 ,0% 0 ,0% 0 ,0%**

**Control Very low 1 %5,0 1 %10,0 0 ,0%**

**Group Low 10 %50,0 4 %40,0 6 %60,0**

**Average 7 %35,0 5 %50,0 2 %20,0**

**Good 2 %10,0 0 ,0% 2 %20,0**

**Very good 0 ,0% 0 ,0% 0 ,0%**

As in Table 7, 4 learners (%20.0) in the experimental group had a very low, 8 (%40.0) low, 8 (%40.0) average, 1 (5.0) learner in the control group had very low, 10 (%50.0) low, 7 (%35.0) average, and 2 (%10.0) low scores.

The findings from the pre-test of the experimental group in terms of gender, 1 (%9.0) girl had very low, 6 (%54.6) girls had low, 4 (36.4) girls had average scores. No any girl learner had a very good score. 3 (33.3) boys had very low, 2 (22.3) had low, 4 (44.4) average scores.

The post-test scores by the control group concerning owing any of the devices do not indicate to a different effect.

The ppermanent-test results by the control group concerning owing any of the devices do not indicate to a different effect.

1 (%10.0) of the girls in pre-test control group had very low, 4 (%40.0) low, 5 (%50.0) average scores. 6 (60.0) of the boys in the pre-test control group had low, 2.(%20.0) average, and 2 (10.0) had good scores.

At this point, it is clear that in terms of pre-test success scales, the control group showed a higher performance than the experimental group. In terms of gender, in both groups girls exhibited a lower performance than boys.

**Table 8. The post-test performance scales of experimental and control groups**

**Test Groups Scales Total Gender**

**Girls Boys**

**N % N % N %**

**Post-test Experimental Lowest 0 ,0% 0 ,0% 0 ,0%**

**Group Low 0 ,0% 0 ,0% 0 ,0%**

**Average 0 ,0% 0 ,0% 0 ,0%**

**Good 6 %30,0 5 %45,5 1 %11,1**

**Highest 14 %70,0 6 %54,5 8 %88,9**

**Control Lowest 0 ,0% 0 ,0% 0 ,0%**

**Group Low 1 %5,0 1 %10,0 1 ,0%**

**Average 7 %35,0 3 %30,0 4 %40,0**

**Good 7 %35,0 3 %30,0 4 %40,0**

**Highest 5 %25,0 3 %30,0 2 %20,0**

As can be seen in Table 8, 6 (%30.0) learners of the experimental group had good, and 14 (%70.0) had the highest scores in the post-test. 1 (%5.0) of the control group had low, 7 (%35.0) average, 7 (%35.0) good, and 5 (%25.0) highest results.

.

In the same test 5 (45.5) girls scored good and 6 (%54.5) the highest. 1 (%11.1) of the boys had good, 8 (%88.9) the highest scores.

The result of the analysis reflects that 1 (%10.0) girl had low, 3.(%30.0) average, 3 (%30.0)good and 3 (%30.0) the highest scores. 4 (%40.0) of the boys had average, 4 (%40.0) good, and 2 %20.0) the highest scores.

When the performance scales are overviewed, it can be seen that the control group had a lower performance thatn the experimental group.

In terms of gender, it is observed that boys had higher scores compared to girls.

**Table 9. Performance scales of experimental and control groups in permanent-test success**

**Test Groups Scales Total Gender**

**Girls Boys**

**N % N % N %**

**Permanent Experimental Lowest 0 ,0% 0 ,0% 0 ,0%**

**Test Group Low 0 ,0% 0 ,0% 0 ,0%**

**Average 1 %5,0 1 %9,1 0 ,0%**

**Good 7 %35,0 4 %36,4 3 %33,3**

**Highest 12 %60,0 6 %54,5 6 %66,7**

**Control Lowest 0 ,0% 0 ,0% 0 ,0%**

**Group Low 4 %20,0 2 %20,0 2 %20,0**

**Average 7 %35,0 3 %30,0 4 %40,0**

**Good 6 %30,0 4 %40,0 2 %20,0**

**Highest 3 %15,0 1 %10,0 2 %20,0**

The experimental group permanent-test results, as shown in Table 9, are as; 1 (%5.0) average, 7 (%35.0), and 12 (%60) highest. As for the control group, 4 (%20) had low, 7 (%35) average, 6 (%30.0) good, and 3 (%15.0) highest scores.

The analysis result of the permanent-test reveals that 1 (%9.1) girl participant had average, 4 (%36.4) good, and 6 (%54.5) highest scores. 3 (%33.3) of the boys had good, and 6 (%66.7) the highest scores.

The result of the analysis showed that 2 (%20.0) of the girls had low, 3 (%30.0) average, 4 (%40.0) good, and 1 (%10) the highest score. 2 (%20) of the boys had low, 4 (%40) average, 2 (%20.0) good, and 2 (%20) the ighest scores.

When the performance scales are overviewed, it is seen that the control group had lower scores compared to the experimental group. In terms of gender, in the experimental group boys had higher, but in the control group girls had higher scores.

**Table 10. The t-test Table showing the Pre-test scores by the experimental and control groups**

**Groups N $\bar{X}$ Ss sd t p**

**Experimental 20 42,500 14,001 38**

**Control 20 46,000 14,104 38 -,788 ,436**

The Table does not reveal any significant difference in the Pre-test scores and this is because both groups are in the same level.

**Table 11. The t-test Table showing the Post-test scores by both experimental and control groups**

**Groups N $\bar{X}$ Ss sd t p**

**Experimental 20 88,000 8,335 38**

**Control 20 70,750 12,383 38 5,168 ,000**

The table reveals that the control group had a weaker performance compared to the experimental group.

**Table 12. The t-test Table showing the permanent test results by both the experimental and control group**

**Groups N $\bar{X}$ Ss sd t p**

**Experimental 20 84,250 8,626 38**

**Control 20 62,500 16,741 38 5,165 ,000**

It can be seen in the Table that the control group were weaker than the experimental group in the permanent-test.

**Table 13. The t-test Table showing Pre/Post-test results by both the experimental and control groups**

**Groups Test N $\bar{X}$ Ss sd t p**

**Experimental Pre-test 20 42,500 14,001 19 -13,379 ,000**

**Post-test 20 88,000 8,335**

**Control Pre-test 20 46,000 14,104 19 -5,657 ,000**

**Post-test 20 70,750 12,383**

The Table reveals a significant difference between the Pre/Post-test average scores (p<0.01).

**Table 14. The Pre/Permanent-test results by both the experimental and control groups**

**Groups Test N $\bar{X}$ Ss sd t p**

**Experimental Pre-test 20 42,500 14,001 19 -13,379 ,000**

**Permanent- 20 84,250 8,626**

**test**

**Control Pre-test 20 46,000 14,104 19 -3,264 0,004**

**Permanent 20 62,500 16,741**

**test**

As shown in Tble 14, there is a significant difference (p<0.01) between the averages of the Pre/Permanenet-test scores.

**Table 15. The Post/Permanent-test results by the control group**

**Groups Test N $\bar{X}$ Ss sd t p**

**Experimental Post-test 20 88,000 8,335 19 1,370 0,186**

**Permanent- 20 84,250 8,626**

**test**

**Control Post-test 20 70,750 12,383 19 1,849 0,080**

**Permanent 20 62,500 16,741**

**test**

A significant difference between the post-test scores is observed in the Table (p<0.01). In the post/Permanent-test results A significant difference is observed (p<0.01) in the Post-Permanent-test results.
